# Detecting bone lesions in the emergency room with medical infrared thermography

**DOI:** 10.1186/s12938-022-01005-7

**Published:** 2022-06-13

**Authors:** Wally auf der Strasse, Daniel Prado Campos, Celso Júnio Aguiar Mendonça, Jamil Faissal Soni, Joaquim Mendes, Percy Nohama

**Affiliations:** 1 CPGEI - Universidade Tecnológica Federal Do Paraná, Avenida Silva Jardim, 3165, Rebouças, Zip code 80230-901 Curitiba, Brasil; 2 PPGCS - Pontifícia Universidade Católica Do Paraná, Curitiba, Brasil; 3 Faculdade de Engenharia, Universidade Do Porto, Porto, Portugal; 4 INEGI, Porto, Portugal; 5 PPGTS - Pontifícia Universidade Católica Do Paraná, Curitiba, Brasil

**Keywords:** Infrared thermal imaging, Bone fracture, Screening temperature, Bone lesion identification, Diagnostic tool, Emergence room

## Abstract

**Introduction:**

Low- to high-energy impact trauma may cause from small fissures up to extended bone losses, which can be classified as closed or opened injuries (when they are visible at a naked eye).

**Objective:**

The aim of this study was to investigate the feasibility of clinical diagnosis of bone trauma through medical infrared thermography, in a hospital emergency room.

**Methods:**

Forty-five patients with suspected diagnosis of bone fracture were evaluated by means of medical infrared images, and the data correlated with the gold standard radiographic images, in the anteroposterior, lateral, and oblique views, at the orthopedic emergency department. The control group consisted of thermal images of the contralateral reference limb of the volunteers themselves. Data were acquired with a medical grade infrared camera in the regions of interest (ROIs) of leg, hand, forearm, clavicle, foot, and ankle.

**Results:**

In all patients evaluated with a diagnosis of bone fracture, the mean temperature of the affected limb showed a positive difference greater than 0.9 °C (towards the contralateral), indicating the exact location of the bone trauma according, while the areas diagnosed with reduced blood supply, showed a mean temperature with a negative variation.

**Conclusion:**

Clinical evaluation using infrared imaging indicates a high applicability potential as a tool to support quick diagnosis of bone fractures in patients with acute orthopedic trauma in an emergency medical setting. The thermal results showed important physiological data related to vascularization of the bone fracture and areas adjacent to the trauma well correlated to radiographic examinations.

## Background

Bone fractures are considered common orthopedic trauma in emergency room departments in a hospital environment, they can result from high-energy trauma such as car accidents, falls from heights, injurie due to shot guns, or otherwise from low-energy trauma caused by sports injuries or bone diseases such as osteoporosis, and bone tumor [1]. Traumas such as cracks in bone tissue caused by excessive exercise or repeated bone impacts, are called stress fractures [2]. Closed fractures tend to cause an increase in temperature at the bone lesion site. These thermal changes are originated by increased blood flow around the lesion, as observed at the fracture site, already highlighted by previous studies [3]. However, the temperature may also decrease, depending on the severity of the injury, if the regenerative process stops or if blood flow reduces [4].

In the initial screening of injuries performed by orthopedic surgeons, the gold standard is the diagnostic evaluation through medical X-ray imaging. However, many injuries are not apparent in the first weeks of bone trauma, such as stress fractures, which only show traces of damage to the cortical bone, 1 to 2 weeks after the traumatic event [5, 6]. Yet another difficult fracture to diagnoseis radial bone fracture in pediatric patients, where the X-ray examination needs to be repeated after 1 or 2 weeks of trauma for confirmation [7]. In the trauma emergency room, novel technologies point to the importance of supporting the diagnosis of the injury and involvement of tendons and soft tissues adjacent to the fracture site.

Diagnostic exams by infrared imaging have already been used in other medical specialties as a diagnostic imaging exam for medical screening. The authors Saxena et al. 2020, performed dynamic thermographic capture using an external cooling stimulation of the neck region in the carotid artery passage, and then correlate with Doppler ultrasound examination with the objective of evaluate carotid stenosis. The accumulation of atheromatous plaque inside the artery made it difficult for blood to flow to the neck and face, thus causing a decrease of the temperature.

IRT may be also useful in early medical screening of abnormalities in the vascularization of the superficial breast tissue to detect cancer nodules. The authors Rani [8] and [9] through automatic segmentation of thermal imaging and correlation with mammography examination (breast radiography), point out thermography as an imaging technology supporting early breast cancer screening. Therefore, bimodal analysis of images has diagnostic importance, in the evaluation of medical images using machine learning and development of specific algorithm parameters for correlation of medical images [10].

Thermography captures the thermal changes expressed by the cutaneous microcirculation, and inflammatory response presented by the skin in acute trauma. Arterioles and dermal venules are composed of endothelial cells, connective tissues (collagen, elastin) and smooth muscle cells that allow vasoconstriction and vasodilation of veins and peripheral blood capillaries, presenting local vascular effects on bone lesions [11, 12]. In view of this, the authors [13] investigated the potential of imaging using infrared thermography as a diagnostic technology to rule out bone fractures in a pediatric trauma emergency department. The researchers analyzed thermal images of 145 children who had suffered a traumatic injury and underwent X-ray examination. In the bimodal correlation of diagnostic images, infrared thermography demonstrated a sensitivity of 91% and specificity of 88% in the detection of bone fracture. In addition, a negative predictive value of 95% was reported. Thus, the authors conclude thermal images are effective in the exclusion of pediatric fracture in cases of acute trauma. Likewise, the authors of [14] evaluated the use of diagnostic infrared imaging to assess children who had gait difficulties, with the initial diagnosis of acute undifferentiated claudication. The authors pointed out the thermal examination as an important technology to support the screening of bone lesions. They also highlight the infrared thermography exam presents a diagnostic potential in the detection of bone fractures before to be visible on radiographic images.

Just after the fracture episode, there is a vascular interruption, and an immediate inflammatory response at the trauma site close to the injury [15]. The acute inflammatory process causes an increase in the cytokines proteins that accumulate at the site of injury, which increases the metabolism and the temperature of surrounding tissues [3]. Subsequently, there is a greater recruitment of mesenchymal stem cells and subsequent differentiation into chondrocytes, followed by vascular ingrowth and intensification of neo-angiogenesis. The cells stimulate the growth of blood vessels in the periosteum, the production of cartilage, and increase the action of osteoblastic cells to initiate bone repair [16]. The inflammatory process and changes in blood perfusion causes a change in the thermal pattern visible at the skin through medical infrared thermography (IRT) [4, 15]. In view of these aspects, the use of IRT has been increasingly investigated, highlighting diagnostic images of trauma in the extremities of upper limbs in pediatric patients [7]. Likewise, Vardasca investigated the temperature distribution symmetry patterns of the upper and lower extremities, by using IRT to discriminate and establish thermal profile in healthy people for correlation with injuries [17].

Di Benedetto et al. observed regions with decreased temperatures in non-severe traumatic injuries [18]. Likewise, other investigations use IRT in patients diagnosed with bone nonunion in bone elongation process [19, 20], or in the assessment of focal thermal changes in vertebrae with increased bone resorption after drug administration of bisphosphonates in the treatment of osteoporosis of the lumbar spine [21].

The authors [22] also performed IRT in a trauma department, at an emergency unit of a Spanish hospital. A total of 133 radiographs of trauma in forearm bones were correlated with thermal images acquired in pediatric patients and the results demonstrated a sensitivity of 91% and specificity of 81% in the examination. Thus, the authors also suggest IRT as a promising diagnostic method for identifying bone fractures.

The technological advances achieved by infrared cameras in recent years have resulted in an improved diagnostic capability and greater confidence, which has increased the sensitivity and spread the method's employability in various clinical specialties [23, 24] and having found wide acceptance in the medical community [12]. Efficiency, combined with the lowest comparative financial cost of the magnetic resonance imaging (MRI) and computer tomography (CT) technologies, combined with the safety of image acquisition as it does not emit ionizing radiation, makes thermography an auxiliary tool in diagnostic imaging medicine [23].

However, the applicability of IRT is limited for monitoring fractures in apparent bones, which are closer to the skin, otherwise for bones covered with a large layer of muscle tissue, such as the femur bone, it is not possible to perform a diagnostic by thermography.

Given the above, this study investigated the feasibility of using IRT in the immediate assessment of acute fractures in adult patients. For this purpose, images were obtained from apparent bones of forearm, hands, clavicle, leg, ankle, and feet that were analyzed and correlated with conventional X-ray images, in a hospital emergency department, as to support the clinical diagnosis of bone trauma.

## Results

Six groups of bone fracture diagnoses were evaluated through thermographic images, 8 in the leg bones, 14 in the hands, 10 in the forearm bones, 4 in the trunk, 6 in the feet and 3 in the ankle, as shown in Table 1.

**Table 1:** Patient demographic

Diagnostic group						
Demographic parameters	Leg	Hand	Forearm	Trunk	Foot	Ankle
Sex						
Male	7	10	3	2	4	2
Female	1	4	7	2	2	1
Side fracture						
Right	4	9	6	3	1	2
Left	4	5	4	1	5	1

As the acquisition of thermographic images in the trauma emergency was performed during the COVID-19 pandemic, on admission to the hospital, all patients underwent thermal screening by an infrared sensor to check for fever.

**Fig. 1:**
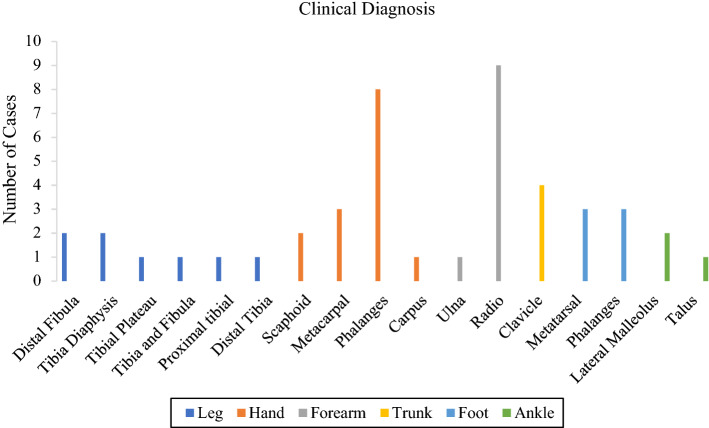
Sample size per body segment diagnosed with bone fracture

In total, 45 patients were evaluated in an orthopedic emergency department, with a diagnosis of thermal imaging with the presence of hot spots indicative of suspected bone fracture confirmed in the initial screening by X-ray image, as shown in Fig. 1.

Regarding the 8 legs fracture diagnosis, thermographic images of the contralateral limb were not obtained in two patients with tibial fractures, as the evaluated patients entered the hospital on a medical rescue ambulance stretcher, which prevented the removal of the clothing that covered the contralateral leg. Likewise, in seven thermographic evaluations in hand trauma, seven in the forearm, two fractures in the feet and one in the ankle region, the same problem occurred. In addition, most of them present strong painful symptoms, so compulsory manipulations of the body were avoided.

In the analysis of the mean temperatures obtained from thermal images of the eight volunteers acquired in the lower limbs, the data showed that in the fracture which occurred in the proximal tibial (plateau), the temperature immediately besides the fracture was higher when comparing to the distal portions and fibula. It was highlighted in the observations of the thermal images that in the case of double diaphyseal bone fracture (tibia and fibula), the thermal data denoted an important comparative temperature change, with a mean difference of 4.5 °C when compared to the contralateral limb.

**Fig. 2:**
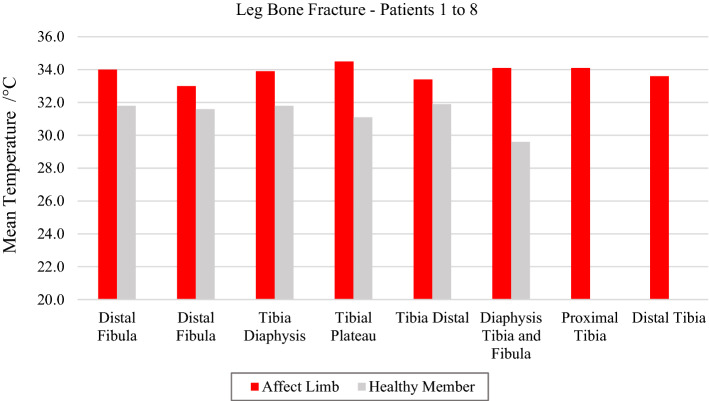
Mean temperatures in tibial and fibular fractures and contralateral reference limb

**Fig. 3:**
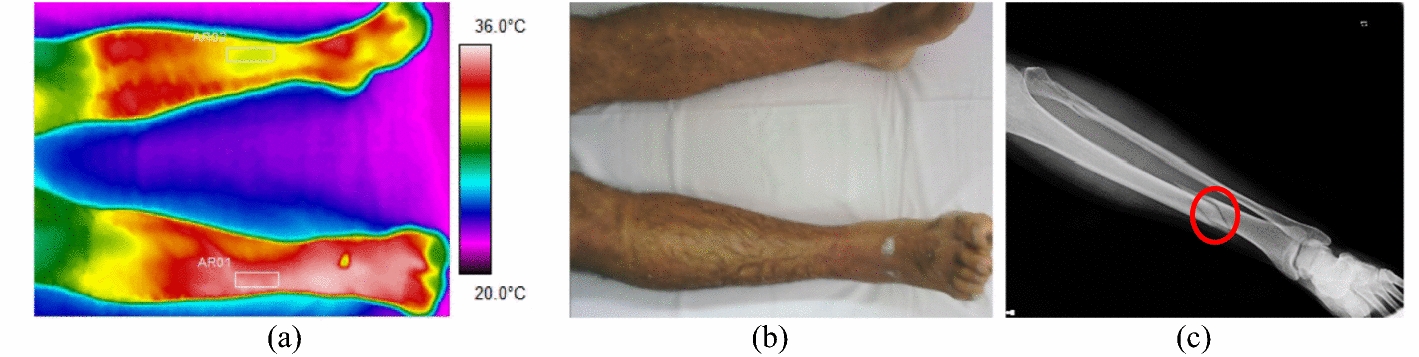
Fracture of the right tibia: **a** infrared image of the patient's lower limbs with the delimitation of the ROIs; **b** photograph for comparative analysis; **c** Radiographic image of the fracture

The difference between the average temperature in the affected limb towards the contralateral ranges from 1.4 to 3.4 °C (volunteer 4, at tibial plateau), according to data shown in Fig. 2 and corresponding X-ray and visible images, Fig. 3.

The analysis of the images involving fractures of the segment of the hands showed differences in the mean temperatures according to the time of occurrence of the trauma. Regarding the metacarpal and carpal bones (volunteers 10, 16, 18 and 21), higher mean temperatures were measured in the assessment of acute trauma; however, in the assessment of metacarpal fracture 3 weeks after the occurrence, the data demonstrated thermal normalization, showing equal temperatures (28.3 °C) in both limbs. In evaluated traumas of the scaphoid bone (volunteers 9 and 12), considered fractures that require longer healing time due to its joint mobility, the mean temperature was higher than 33.0 °C.

In phalanges trauma assessments and according to their location in the portion of the fractured finger, the average temperature range showed great thermal variability, with values between 28.7 °C and 35.0 °C; the highest value was in the proximal phalangeal fractures.

Patient number 17 entered the emergency room with an open fracture and amputation of the 4th proximal finger of the right hand. Due to the wound and loss of tissue at the injury site, the hand has a lower mean temperature than the contralateral one.

In general, the temperatures in the affect limb are higher than the ones observed in the healthy one. However, there were two cases of fracture of the phalange proximal portion (ring and middle ring) that showed a lower temperature compared with the contralateral. The reason for this seems to be originated by the patient that used the healthy hand to grasp the affect hand to reduce the feeling of pain and eventual bleeding. This natural behavior led to an increasing of the temperature in the health region (due to muscular strength) and lowering the one affected (due to vascular network constraining).

**Fig. 4:**
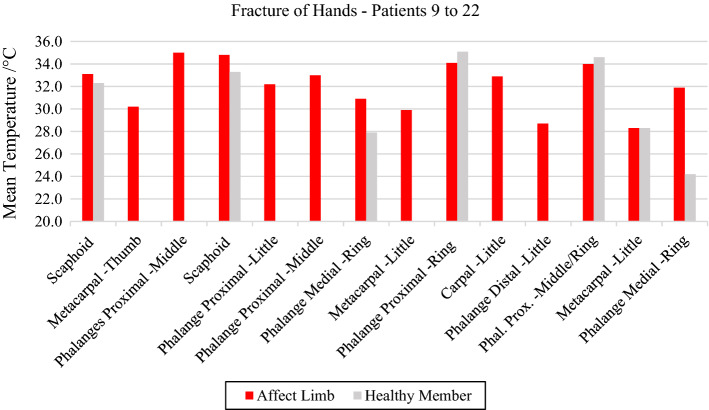
Mean temperature in the ROIs corresponding to the fractures and contralateral in bones of the hand

**Fig. 5:**
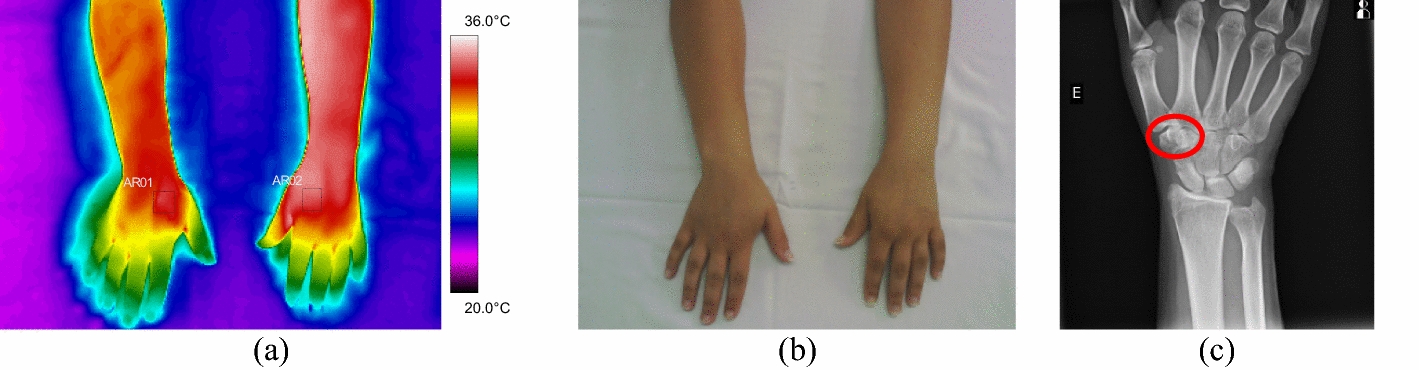
Left scaphoid bone fracture: **a** infrared thermogram of the volunteer's lower limbs with delimitation of the ROIs; **b** a photograph for comparative analysis; **c** corresponding radiography

On the other hand, a large thermal change was observed in the injury of volunteer 22, due to a total fracture of the medial phalanx and tearing of the tendon of the fourth finger, requiring reconstructive surgery, as shown in Fig. 4 and the corresponding X-ray and visible images, Fig. 5.

**Fig. 6:**
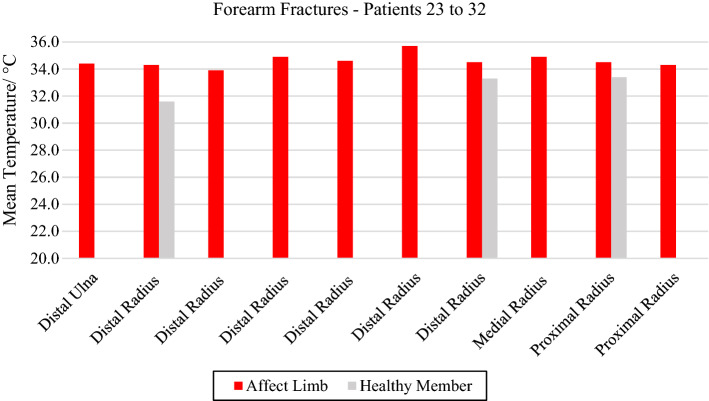
Mean temperature in fractures in the bones of the forearm and contralateral reference limb

**Fig. 7:**
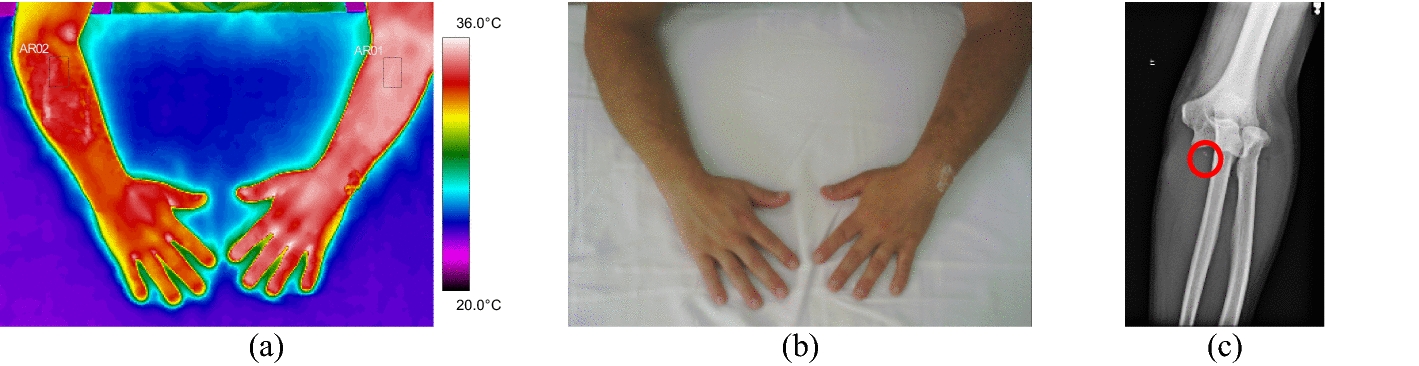
Proximal fracture of the right radius bone: **a** infrared image of the volunteer's upper limbs with the delimitation of the ROIs; **b** photograph for comparative analysis; **c** radiographic image for diagnostic correlation

In the ten forearm fractures, the most common trauma was the distal portion of the radius bone. All evaluated volunteers entered the hospital after a fall from a level or a car accident, with intense pain symptoms. In some patients, it was not possible to obtain a thermal image of the contralateral limb due to the difficulty of removing the cloths. Mean temperature in the affected forearm ranged from 33.9 to 35.7 °C. Differences in mean temperature compared to the healthy limb were higher than 1.0 °C, according to data shown in Fig. 6 and the corresponding X-ray and visible images, Fig. 7.

**Fig. 8:**
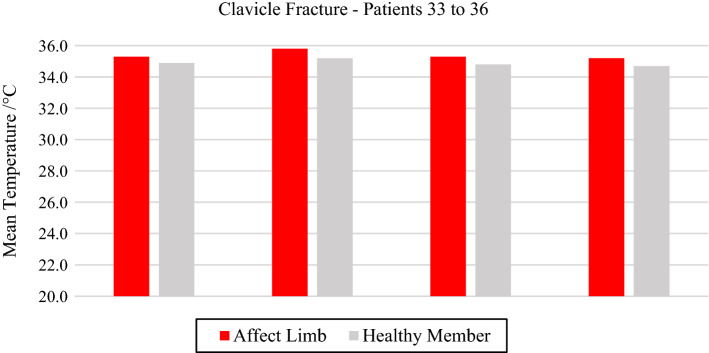
Mean temperatures in fractures of the clavicle bone and contralateral reference limb

**Fig. 9:**
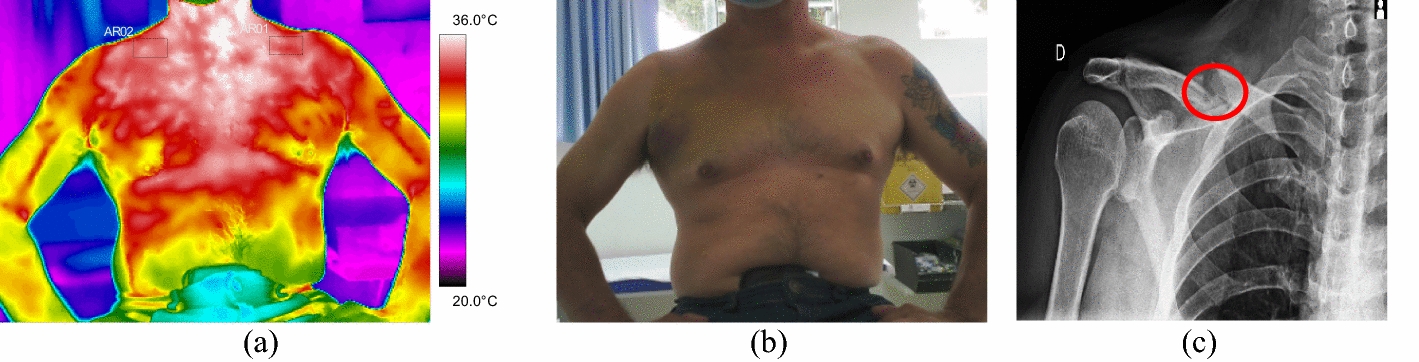
Right clavicle fracture: **a** infrared image of the volunteer's lower limbs with the delimitation of the ROIs; **b** photograph for comparative analysis; **c** radiographic image for diagnostic correlation

All the trunk fractures were provoked by fall and occurred on the right side of the body hemisphere. They were diagnosed in the clavicle bone, with mean temperature values above 35.0 °C. Volunteer 34 had a fracture with displacement, with strong painful symptoms and bone prominence on clinical palpation, according to data shown in Fig. 8 and the corresponding X-ray and visible images, Fig. 9.

The evaluated traumas in feet occurred mainly in the bone portions of the toes. For two volunteers, there was no possibility to acquire comparative images of the contralateral limb.

**Fig. 10:**
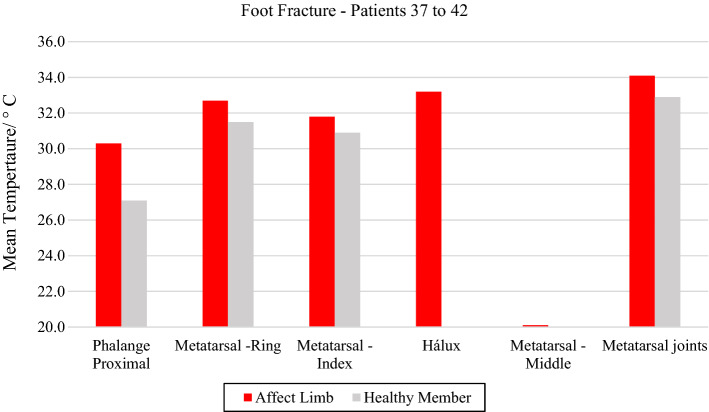
Mean temperatures in foot fracture bone and contralateral reference limb

**Fig. 11:**
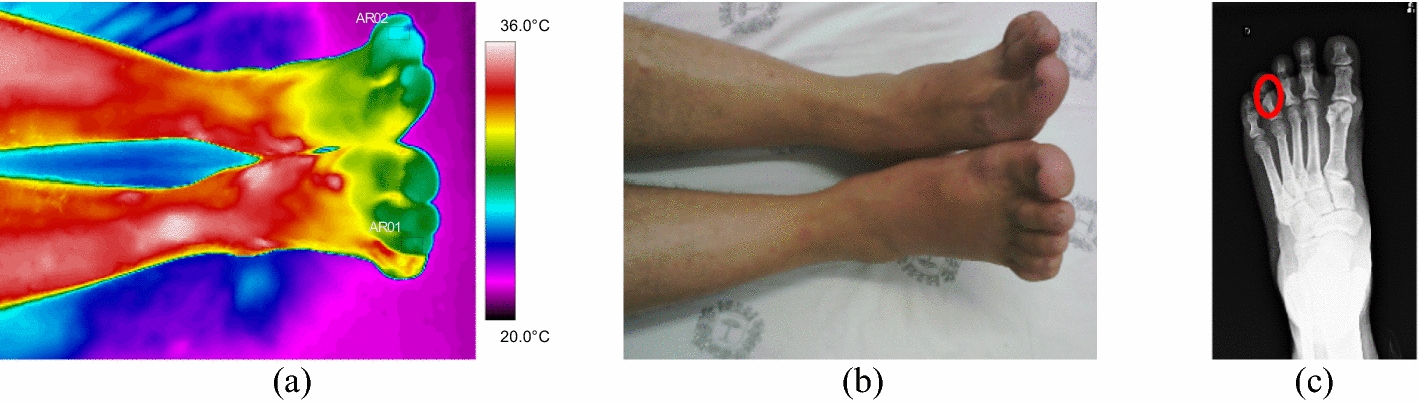
Left foot phalange fracture: **a** infrared image of the patient's lower limbs with the delimitation of the ROIs; **b** photograph for comparative analysis; **c** diagnostic correlation radiographic image

Volunteer 40 had an open fracture of the big toe, in the distal portion of the phalanx, with more accentuated thermal change (33.2 °C), volunteer 42 also had a Lisfranc fracture, in the middle foot, with rupture of the three tarsometatarsal joints, exhibiting high temperature (34.1 °C). Patient number 41, on the other hand, denoted a very cold temperature in the distal portion of the bone phalanx of the third finger (20.1 °C), as the diagnostic evaluation was carried out 3 days after the occurrence of the trauma, as shown in Fig. 10 and the respective radiographs and visible images, in Fig. 11.

The thermographic image, Fig. 11a, shows a thermal change in the region affected by the trauma, also visible in the X-ray, where there is a small trace of bone fissure in the medial phalanx of the left foot, Fig. 11c. However, the trauma region presents a lower temperature which maybe originated by several factors: the patient has lupus erythematosus—a peripheral vascular disorder that causes by vasoconstriction; due to the trauma, the patient applies less weight to the left foot; the positioning of the toes (left and right) are not exactly with the same orientation towards the camera (the ROIs should be as perpendicular as possible); the space/contact to the other fingers.

**Fig. 12:**
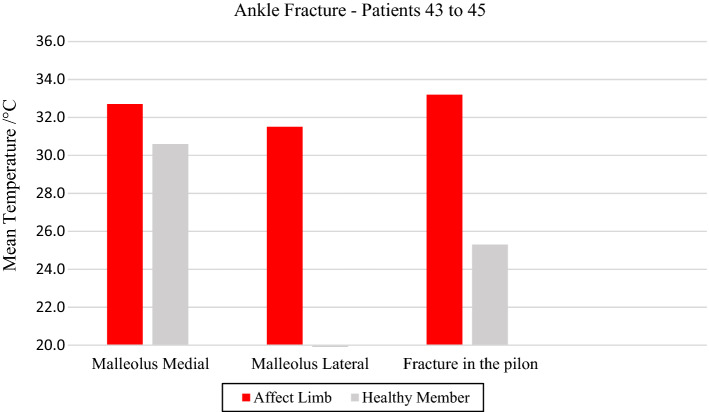
Mean temperatures in ankle fracture bone and contralateral reference limb

**Fig. 13:**
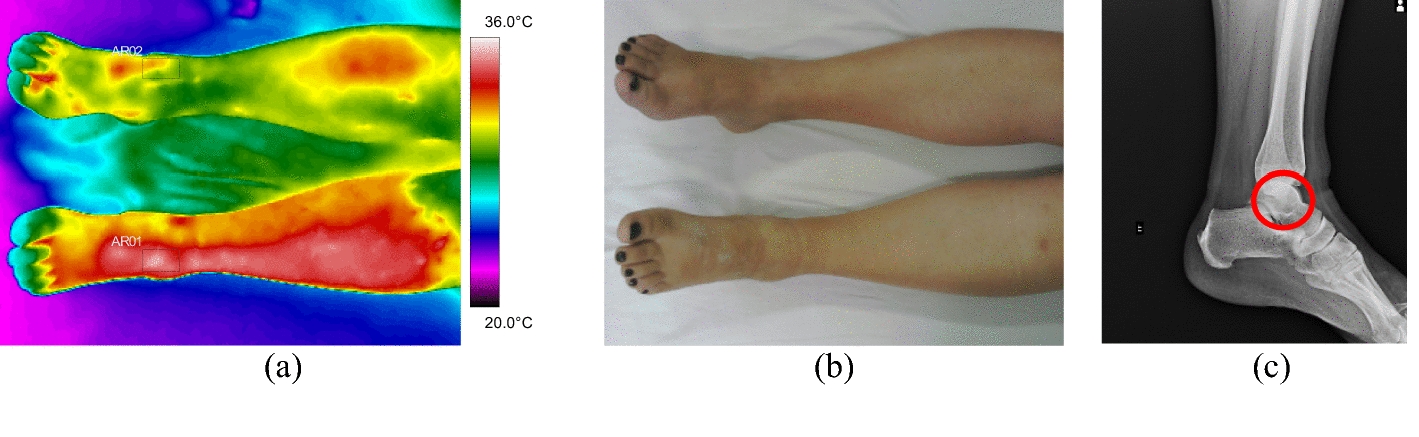
Left malleolus fracture: **a** Infrared image of the patient's lower limbs with the delimitation of the ROIs; **b** photograph for comparative analysis; **c** diagnostic correlation radiographic image

The thermographic images obtained from the ankle fractures were evaluated in the medial malleolus of volunteer 43 and in the lateral malleolus of the fibula in volunteer 44. Bone fracture in the pylon region of the tibia distal portion presented by volunteer 45, showed a high thermal difference (7.9 °C) at the trauma focal point, compatible with the radiologic images and strong painful symptoms expressed by the volunteer, Fig. 12 and the corresponding X-ray and visible images, Fig. 13.

## Discussion

Acute bone fractures present hyperthermia and intense inflammatory response within 24 h after trauma [25], providing a predictive indication of bone injury by means of the infrared images, due to the relevant increasing in the measured temperature in the lesion focal point, and on adjacent areas of the assessed body segment [7]. The detection of the highest temperature area favors the targeting of radiographic image acquisition. Indeed, the thermal pattern reflects the underlying physiology of the affected limb by increasing / decreasing the peripheral blood flow regulated by the autonomic nervous system [11, 13].

Regarding the analysis of the 10 thermal images of the volunteers investigated with forearm fractures, it was observed that the temperature of the radius and ulna showed temperature differences between 1.0 and 2.7 °C, denoting thermal changes compatible with bone fracture diagnostic. These data were corroborated by the findings of the authors [26], who investigated changes in temperature on the day of trauma and during the healing of the fracture in the forearm in a clinical follow-up of 19 pediatric patients. The authors observed differences of up to 2.8 ºC in the first week of the fracture, as measured in our study for volunteer 24 with radiographic diagnostics of distal radius fracture.

The study [27] developed by Charters also demonstrated that the use of IRT was useful in the detection of fractures of the radius and distal ulna in pulses of 67 children. The results analysis indicated that from a total of 34 thermal images 31 of them confirmed the fracture diagnostic performed by X-ray imaging. They presented a temperature difference at the fracture focal point higher than 1 °C when compared to the control limb used as thermal reference. Thus, showing the feasibility of thermal imaging as a diagnostic tool in children in the emergency room environment, with diagnostic sensitivity of 96.8% when compared to X-rays, and up to 96.7% if compared to clinical examination.

In view of the investigations in pediatric patients, the results evaluated in 105 children with wrist fractures stand out that bone trauma is often not visible in the first radiographic images. The authors [15] evaluated the patients through infrared diagnostic images, with screening in diagnoses of sprain and bone fracture, and proposed an algorithm for differentiating the two diagnoses, orthopedic trauma as a screening or adjunct to clinical diagnosis in a hospital setting.

In the unique study with adult volunteers evaluating forearm bone fractures, the researchers [3] investigated fractures in the distal portion of radius bone in the orthopedic emergency environment, with imaging on the trauma day and subsequent follow-up evaluations. The results also showed thermal differences greater than 0.6 °C, compared to the reference contralateral limb. The authors also pointed out the feasibility of thermographic examination in the orthopedics clinical practice, as an excellent method of monitoring the evolution of the bone healing process, strongly corroborating our results in the evaluation of forearm traumas and the diagnostic.

Regarding the thermal responses verified in hand bones, the results showed differences in the mean temperature measured in the phalanges, in the diagnosis of bone fracture with amputation of the distal portion of the fourth finger (34.1 °C), verified in volunteer number 17, and increase of mean temperature in a scaphoid bone fracture (34.8 °C), in volunteer 12. The measured temperatures had denoted to be elevated for this body segment, considering that body extremities tend to be lower due to the decrease in vascularization.

Infrared images of the hand were evaluated by Snekhalatha et al. [28] to detect rheumatoid arthritis, based on the heat distribution. In healthy people, automatic segmentation of abnormal regions of the hand of patients with arthritis, was performed using fuzzy algorithm and expectation maximization (EM) algorithm.

The results showed a 0.96 °C increase on temperature in the hand region of volunteers with arthritis compared to healthy volunteers. In the present study, 14 patients with hand fractures diagnostics show an increase of more than 1 °C in mean temperature difference to the contralateral. Depending on the injury severity, an eventual displacement or rotation of the bone fragments may happen, as verified in volunteer 22, in the fracture diagnostics of the proximal phalanx, thermal differences were even higher (7.7 °C).

The researchers [29] also carried out investigations of infrared thermal imaging in hands and upper limbs. The results strengthen the applicability of infrared imaging exams, presenting it as a safe and innocuous technology, providing additional data to be added to the immediate diagnostics and the patient's clinical follow-up.

In the same sense, the researchers [17] investigate thermal symmetry of the body segments of arms, hands, legs, and feet in 39 adult male volunteers. The results showed that temperature asymmetry can be significant in musculoskeletal disorders, with unilateral manifestation in the affected limb, while in the extremities (hands and feet) healthy individuals have a maximum difference of 0.4 °C.

Another study of medical thermography in bone lesions of the lower limbs is the research by [30] who analyzed extraction algorithms to determine predictive characteristics for detecting orthopedic trauma abnormality, based on the Euclidean distance, which is the metric distance between two points, between the average pixel and all other image pixels. These authors proposed a Euclidean distance for color image segmentation algorithm based on abnormality of thermographic examinations of arthritis, stress fracture, long bone fracture, and ankle injury. This research demonstrated that the Euclidean distance applied in the thermal images analysis can be a parameter for detecting abnormalities in the lesions studied in apparent bones. These results corroborate our analysis of the 45 thermograms, as thermography allows the exact determination of the fracture site in the images, which facilitates the correct positioning of radiographic image.

Still referring to the applicability of IRT as a supporting tool for screening and early diagnosis in the hospital emergency department, the researchers [31] evaluated thermograms of 110 adult patients of both genders, acquired in foot, leg, thigh, hand, forearm and arm, ankle, knee, wrist, elbow, and shoulder, for the occurrence of acute trauma. The predictive values in the ROIs, specifically evaluated in bone fractures, stood higher than 0.9 °C when comparing the affected to the contralateral limb.

A diagnostic bone fracture results in disruption of vascularity, an acute inflammatory response and in some cases can lead to compartment syndrome. The regions of interest for thermal analysis include the lesion and surrounding tissues. In the analyses performed, differences of temperature above 0.9 °C were indicative of a suspected bone fracture, to be confirmed by radiographic examinations. This cut-off will gain robustness as larger samples are becoming available, namely with different types of fractures (simple, exposed, and incomplete). The authors [31], also pointed in their analyses as an indication of bone fracture the value of 0.9 °C, in comparison with healthy contralateral limb or the same limb surrounding areas if the first is not available.

The obtained results present similar characteristics to those found in the investigation presented here, in which thermal differences greater than 0.9 °C were observed in diagnostics of legs’ bone fractures, forearms, hands, feet and ankles, in the comparison between the images of the volunteers' fractured portion of the bone and the healthy ones. However, the four evaluated volunteers with clavicles fractures did not present the same thermal difference between the images of the fractured and the healthy portion, denoting the difference of 0.6 °C. It is noteworthy that the investigated clavicular fractures presented mean temperature values higher than 35.0 °C in all evaluations, with very prominent hot spots on the thermal images, which could be correlated to radiological imaging and anatomical palpation.

The findings of researchers [31] reinforce our main results about the feasibility of thermographic cameras for clinical medical use in the hospital emergency department. Examinations using infrared images as additional diagnostic technology, is sensitive to orthopedic trauma and bone fractures, providing the identification of the anatomic site with abnormal thermal changes to aid the correct diagnosis, avoiding the realization of a second radiological image (due to an error in the positioning of the patient).

### Limitations

Despite the thermal difference from the affected limb towards the contralateral being the most adequate protocol, for patients with severe pain or fractures, it was not possible to remove the garments or positioning them to capture the thermographic images of the contralateral. In those cases, the surrounding areas may be used as a reference.

It is important to highlight that the thermal images of clavicle and ankle fractures must be observed with caution, due to the small number of volunteers with fractures in those segments; therefore, the data cannot be extrapolated as thermal reference for clinical diagnostics.

In this study all the patients suffered from a fracture. It would be useful to add a control group with soft tissue wounds for comparison. However, due to COVID, only urgent cases were admitted to emergency department. Thermal differences above 0.6 °C are considered an indication of vascular abnormality, which can be originated by sprains, stress fractures or incomplete closed fractures. In the investigation carried out by Reed et al. [32], it was possible to make the thermal differentiation of diagnoses of fractures and wrist sprains with higher values in the first.

Another relevant limiting factor is the reference studies with medical thermography investigations focused on the assessment of pediatric patients in hospital emergency departments, with few investigation studies being conducted in adult population, making comparative diagnostic analyses difficult.

## Conclusion

Investigation in six body segments of legs, feet, forearm, hands, clavicle, and ankles, of 45 volunteers has demonstrated that infrared imaging is an important complementary diagnostic tool in hospital emergency environments. It may be used as an image modality to support the X-ray gold standard technology for diagnosis and follow-up evaluation of the bone fractures regeneration.

The results show that thermal images indicate the exact location of the traumatic injury, facilitating the correct acquisition of radiological images, as well as the proper positioning of the patient, avoiding repeated exposure to ionizing radiation, with the acquisition of complementary X-rays.

The thermal differences presented in the diagnostics were greater than 0.9 °C in the ROIs in all volunteers surveyed, being indicative of the presence of bone fracture.

Clinical information from the diagnostic reference thermal profile applied to orthopedic trauma, combined with data from gold standard exams, emerge as relevant in the applicability of infrared medical image technology in the hospital emergency settings.

The patients of this study were admitted to the emergency department immobilized on retractable ambulance stretchers and therefore touching and positioning them would cause pain. In these situations, IRT is advantageous as it is contactless, allows images to be taken directly where the patient is, even inside the medical rescue vehicles, therefore speeding up the diagnosis, and can be used widely, even on children due to absence of ionized radiation.

## Materials and methods

### Sample

The sample consisted of 45 adult volunteers, 28 men with a mean age of 34.9 years and 17 women with a mean age of 51.0 years, in emergency care, with clinical suspicion of bone fracture. Images were taken at the emergency department of three hospitals in the city of Curitiba, Brazil, from June 2020 to June 2021.

### Material

The thermal images for bone fractures diagnostics were acquired using a certified medical thermographic camera, Flir model T530 (FLIR^®^ Systems Inc., Wilsonville, Oregon, USA, thermal sensitivity/NETD < 40 mK at 30 °C (42° lens), 1.31 mrad / pixel, allowing the correlation between the thermal and radiological images acquired on the same date. The environment temperature and humidity of the emergency room was monitored using the Mimipa^®^, model MT-24A device.

### Methods

Medical thermal images were performed according to radiographic positioning protocols, in anteroposterior and oblique views, with the patient lying on hospital stretchers in the orthopedic emergency department of the hospital. Thermograms were obtained in the regions of acute bone fractures in apparent bones, in leg body segments (tibia and fibula bones), hands (phalangeal and metacarpal bones), forearm (radius and ulna bones), clavicle (comprising the entire portion bone), feet (phalangeal, tarsometatarsal and metatarsal bones) and ankle (talus bone and medial and lateral malleolus).

The camera was positioned at a fixed distance of 40 cm from the bone lesion and kept perpendicular to the assessed body region. Thermographic examinations were performed after the patient acclimatization period to the temperature of the emergency room 21 ºC and 50% RH, no direct sunlight on the subject, in accordance with the international thermography standard: SS 582–1: 2020 [33]. Data were processed through Flir Tools 6.4 software and exported to be analyzed in the MATLAB R2021a for construction of thermal histograms, Boxplot of the medians and temperature dispersion graphs of the evaluated region. All the patients performed a radiographic examination in the same day as the thermal images.

To analyze the thermal changes, the contralateral limbs of the patients were analyzed as a thermal reference for normal temperature of a healthy limb (without fracture) according to the research by [12, 34], and thermal imaging guidelines by [11].

**Fig. 14:**
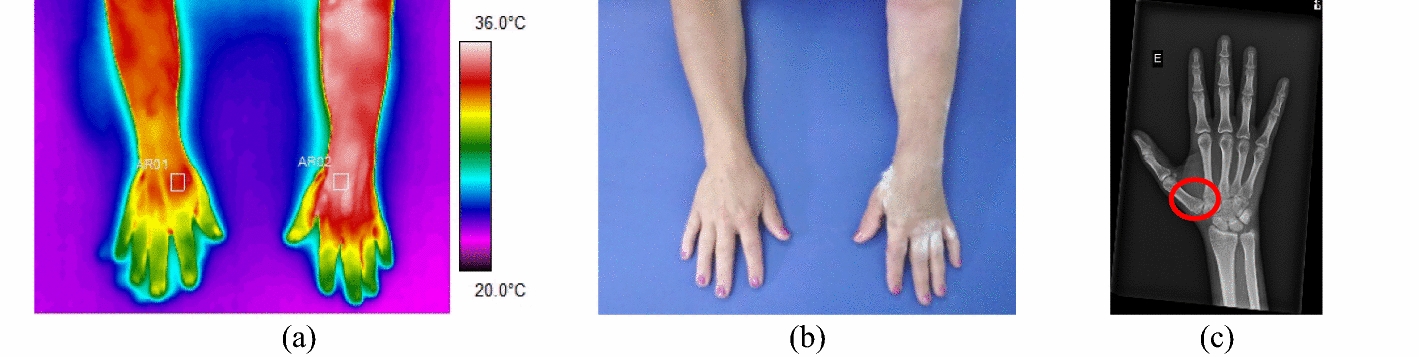
**a** Shows the thermal image of an acute fracture of the left hand (scaphoid bone) and contralateral limb, with the delimitation of (ROI) defined for comparative analysis; **b** visible image of the evaluated limb; **c** shows the radiographic image of the bone trauma

The regions of interest (ROIs) were defined in the evaluated limbs, centered on the trauma areas, covering the adjacent edges and soft tissues. Thermogram analysis was correlated with radiographic images, with particular attention to the vascular changes in trauma site and adjacent areas. The ROIs were defined as rectangles, with dimensions adjusted to the evaluated body segment, centered on the highest temperature portion and adjacent edges, as shown in Fig. 14.

The thermal images were analyzed by comparing rectangular ROIs, with the same size and location, both in the affected limb and in the contralateral and related them to the radiographic images. Thermal data were processed using Flir Tools® 6.4 software to calculate the median temperature for each ROI, and the temperature difference from the affected limb to the corresponding contralateral.

## Data Availability

All data and images are available to editors.

## Abbreviations

ROIs: Regions of interest
IR: Infrared thermography
X-ray: Radiography image
